# Supplementary material for: Molecular characterization of projection neuron subtypes in the mouse olfactory bulb
Source: eLife. 2021 Jul 22;10:e65445. doi: 10.7554/eLife.65445 (PMC8352594; doi:10.7554/eLife.65445)
Supplement: Transparent reporting form [file elife-65445-transrepform.pdf]

eLIFE
1st Floor
24 Hills Road
Cambridge CB2 1JP, UK
P 01223 855340
W elifesciences.org
T @elife

eLife's transparent reporting form

We encourage authors to provide detailed information *within their submission* to facilitate the interpretation and replication of experiments. Authors can upload supporting documentation to indicate the use of appropriate reporting guidelines for health-related research (see EQUATOR Network), life science research (see the BioSharing Information Resource), or the ARRIVE guidelines for reporting work involving animal research. Where applicable, authors should refer to any relevant reporting standards documents in this form.

If you have any questions, please consult our Journal Policies and/or contact us: editorial@elifesciences.org.

Sample-size estimation

- You should state whether an appropriate sample size was computed when the study was being designed
- You should state the statistical method of sample size computation and any required assumptions
- If no explicit power analysis was used, you should describe how you decided what sample (replicate) size (number) to use

Please outline where this information can be found within the submission (e.g., sections or figure legends), or explain why this information doesn't apply to your submission:

Despite single cell RNA sequencing experiments often carried out without biological replicates, we opted for biological triplicates to control for biological variation. For bulk RNA sequencing experiments, we opted for biological triplicates for each injection site as the appropriate number of replicates to use.

Replicates

- You should report how often each experiment was performed
- You should include a definition of biological versus technical replication
- The data obtained should be provided and sufficient information should be provided to indicate the number of independent biological and/or technical replicates
- If you encountered any outliers, you should describe how these were handled
- Criteria for exclusion/inclusion of data should be clearly stated
- High-throughput sequence data should be uploaded before submission, with a private link for reviewers provided (these are available from both GEO and ArrayExpress)

Please outline where this information can be found within the submission (e.g., sections or figure legends), or explain why this information doesn't apply to your submission:

eLife Sciences Publications, Ltd is a limited liability non-profit non-stock corporation incorporated in the State of Delaware, USA, with company number 5030732, and is registered in the UK with company number FC030576 and branch number BR015634 at the address 1st Floor, 24 Hills Road, Cambridge CB2 1JP | August 2014

The snRNA-seq experiment was performed one time using 3 biological replicates from individual mice of the same age, strain, and injection sites (see Figure 2-figure supplement 1).

The bulk RNA-seq experiments were performed on the same day in parallel using 3 biological replicates for each injection site (handling a total of 6 mice) from individual mice of the same age and strain (see FACS plots Figure 1-figure supplement 2).

No technical replicates (from the same mouse) are included in this study.

No outliners were omitted. Figure 2-figure supplement 1 shows that individual biological replicates of snRNA-seq were very similar (as visualized by % of nuclei per cluster).

GEO links are publicly available:
- bulk RNA seq datasets:
https://www.ncbi.nlm.nih.gov/geo/query/acc.cgi?acc=GSE162655
- snRNA-seq datasets:
https://www.ncbi.nlm.nih.gov/geo/query/acc.cgi?acc=GSE162656

Statistical reporting

- Statistical analysis methods should be described and justified
- Raw data should be presented in figures whenever informative to do so (typically when N per group is less than 10)
- For each experiment, you should identify the statistical tests used, exact values of N, definitions of center, methods of multiple test correction, and dispersion and precision measures (e.g., mean, median, SD, SEM, confidence intervals; and, for the major substantive results, a measure of effect size (e.g., Pearson's r, Cohen's d)
- Report exact p-values wherever possible alongside the summary statistics and 95% confidence intervals. These should be reported for all key questions and not only when the p-value is less than 0.05.

Please outline where this information can be found within the submission (e.g., sections or figure legends), or explain why this information doesn't apply to your submission:

For snRNA-seq analysis we used the published Seurat R-packaged version 3 (Butler et al., 2018) according to the instructions of the authors (methods).

For replicates, and for snRNA-seq and simulation integrations, we used the SCT method Hafemeister and Satija, 2019) according to the instructions of the authors (methods).

For differential gene expression (DGE) analysis of snRNA-seq data, we used the glmGamPoi R-package (Ahlmann-Eltze and Huber, 2020) according to the authors instructions (methods).

For gene regulatory network analysis we used the pySCENIC pipeline (Aibar et al., 2017) according to the authors instructions (methods).

(For large datasets, or papers with a very large number of statistical tests, you may upload a single table file with tests, Ns, etc., with reference to sections in the manuscript.)

Group allocation

- Indicate how samples were allocated into experimental groups (in the case of clinical studies, please specify allocation to treatment method); if randomization was used, please also state if restricted randomization was applied
- Indicate if masking was used during group allocation, data collection and/or data analysis

Please outline where this information can be found within the submission (e.g., sections or figure legends), or explain why this information doesn't apply to your submission:

It does not apply.

Additional data files ("source data")

- We encourage you to upload relevant additional data files, such as numerical data that are represented as a graph in a figure, or as a summary table
- Where provided, these should be in the most useful format, and they can be uploaded as "Source data" files linked to a main figure or table
- Include model definition files including the full list of parameters used
- Include code used for data analysis (e.g., R, MatLab)

- Avoid stating that data files are "available upon request"

Please indicate the figures or tables for which source data files have been provided:

Additional information and pipelines for standard snRNA-seq and bulk RNA seq analyses are available at our website (in progress for the final version): https://biologic.crick.ac.uk/OB_projection_neurons

Python and R scripts for gene regulatory network and simulations analyses are available at our GitLab depositories: https://gitlab.com/fleischmann-lab/molecular-characterization-of-projection-neuron-subtypes-in-the-mouse-olfactory-bulb and https://gitlab.inria.fr/acrombac/projection-neurons-mouse-olfactory-bulb.

All data are publicly available under GEO depository (links provided above). We provided snRNA-seq raw data, Cell Ranger count matrices, nuclei metadata (including UMAP coordinates, cluster annotation, subcluster annotation) and bulk RNA-seq raw data.
